# “Should We Take Them or Leave Them?” A Qualitative Study to Understand the Social, Cultural, and Ethical Issues Associated With the Lifecycle Management of Insecticide-Treated Nets in Tanzania

**DOI:** 10.24248/EAHRJ-D-18-00016

**Published:** 2018-11-23

**Authors:** Prince P Mutalemwa, Dennis J Massue, William J Kisoka, Michael A Munga, Bilali Kabula, William N Kisinza

**Affiliations:** a National Institute for Medical Research, Dar es Salaam, Tanzania; b Amani Research Centre, National Institute for Medical Research, Tanga, Tanzania

## Abstract

**Background:**

Insecticide-treated nets (ITNs) are highly effective in reducing morbidity and mortality from malaria. However, it is widely accepted that ITNs – if not re-treated – lose their effectiveness with time and eventually need to be replaced. This study sought to determine the social, ethical, and cultural issues related to the lifecycle of ITNs, which includes net ownership, usage, maintenance, reuse, recycling, disposal, and replacement.

**Methods:**

In this qualitative study, conducted in the districts of Mtwara Rural, Kilombero, and Muheza, Tanzania, we collected information about bed nets, including usage habits, types, treatment status, materials used, brands, acquisition sources, and perceptions thereof. We conducted 23 key informant interviews and 20 focus group discussions with village leaders, other influential people in the community, and district health-care personnel.

**Results:**

ITNs were deemed acceptable and used by most community members in the participating communities. Alternative uses and disposal practices of used bed nets were also common among community members; however, participants had limited knowledge regarding the health and environmental risks associated with these practices. Most participants did not perceive bed net recycling as a sustainable option. Recycling was considered feasible, however, if effective infrastructure for collection and disposal could be established. Poverty was identified as a major driving force towards alternative uses of bed nets. Financial constraints also meant that not all household members were able to sleep under bed nets; pregnant mothers, children under 5 years old, and the elderly were prioritised.

**Conclusion:**

Our findings may inform the National Malaria Control Programme and other stakeholders as they develop country-specific and environmentally friendly bed net replacement strategies. Appropriate strategies will help ensure sustained protection of vulnerable populations against malaria, while considering local social, ethical, and cultural issues related to the recovery of bed nets.

## INTRODUCTION

Globally, about 500,000 deaths result from the over 210 million cases of malaria that occur every year, with 90% of both cases and deaths occurring in sub-Saharan Africa.^1,2^ While malaria control measures include chemotherapy, chemoprophylaxis, and vector control, insecticide-treated nets (ITNs) are a cost-effective preventative measure that can significantly reduce the incidence of malaria and its associated morbidity and mortality. Bed net use is, therefore, a priority for malaria control in sub-Saharan Africa. In Tanzania, where 77.9% of households have at least 1 bed net, an estimated 7.3% of children aged 6 to 59 months have malaria parasitaemia at any given time.^3^

ITNs approved by the World Health Organization Pesticide Evaluation Scheme are considered effective for 3 to 5 years and should be replaced in a timely manner to maximise their efficacy and contribution to malaria prevention. There is a growing awareness of the potential environmental impact of the increasing number of used and discarded ITNs.^4,5^

As it is widely recognised that ITNs lose their effectiveness with time,^4–10^ ITN durability assessments and evaluations must be conducted under various conditions and settings. To address these issues, the World Health Organization released its *Guidelines for Laboratory and Field Testing of Long-Lasting Insecticidal Nets* in November 2005^6^ and *Guidelines for Monitoring the Durability of Long-Lasting Insecticidal Mosquito Nets* in 2010.^7^ These guidelines can be used by country programmes to assess the durability of distributed ITNs to make informed decisions about which ITNs the programmes ought to procure and how often the ITNs should be distributed. At present, however, the general consensus is that ITNs should be replaced every 3 to 5 years.^5–8^

There is emerging evidence that ITNs are often misused,^5,11–14^ although the type and extent of the misuse has not yet been clearly established. Still, documented and anecdotal evidence from monitoring and evaluation activities following ITN distribution campaigns have shown ITNs being used for a range of purposes, including fishing and drying fish in Kenya^12^; protecting a “nursery” (a small crop) in the Solomon Islands^13^; and as ceiling covers, bed covers, room dividers, curtains, tablecloths, and cattle ties in Ethiopia.^14^

While many studies have shown that the most vulnerable household members – particularly, children under 5 years old and pregnant women – are given priority use of available bed nets,^11,14–20^ other studies have reported otherwise in some regions.^11^ Other programme monitoring activities investigating general knowledge about malaria have shown that heads of household often do not know the treatment status of their nets, do not understand why using a treated net is important, and, therefore, they may not know how to prioritise who gets the “best” net.^13,14,17^

Key factors associated with non-use or incorrect use of ITNs include lack of knowledge and misconceptions about the cause of malaria, educational level, type of net, shape of net (ie, conical vs rectangular), perceived efficacy of the net, perceived danger of malaria, perceived discomfort (increased heat), perceived risk, fear of the insecticide, and “saving the net” for future use.^13–16,18,21–23^ The last of these is especially troubling if old nets are no longer effective.

ITNs have been delivered to households through a variety of distribution systems, including the public sector (routine delivery through health facilities, distribution combined with vaccination campaigns, community house-to-house distribution), the private sector (formal and informal markets, social marketing campaigns), and mixed public and private systems. The method through which nets are distributed may affect the success of the campaign, not only in terms of coverage but also in terms of use.^19,20^ Effective and efficient delivery mechanisms – coupled with effective information, education, and communication (IEC) and behaviour change communication (BCC) campaigns – are crucial for ensuring population coverage, appropriate use, and proper maintenance of bed nets.^1,14,17,19,23^ Increasing net usage by vulnerable persons among net-owning households has been a predominant focus of many IEC/BCC activities. The most successful programmes have been “hang-up, keep-up” campaigns, in which volunteers or health-care workers visit households and demonstrate or assist with hanging up bed nets.^19^ If a country was to decide to recollect and recycle used nets, a seamless integration of this activity into existing distribution systems and IEC/BCC campaigns would be paramount.^5^ Any decision made to either encourage or discourage a particular use of a net will need to align with IEC/BCC strategies and new directives to ensure they will not jeopardise existing initiatives.

## METHODS

### Study Design and Data Collection

In this qualitative cross-sectional study, focus group discussions (FGDs) and key informant interviews (KIIs) were used to explore community practices regarding the use or misuse of bed nets; perceptions about net expiry, disposal, and environmental and health risks; and community acceptability of alternative disposal strategies.

### Study Setting

The study was conducted in the 3 districts of Mtwara Rural, Kilombero, and Muheza in Tanzania. The districts were selected based on their high bed net coverage and malaria rates.^3,25–28^

Mtwara Rural (Latitude: 10°16’ S, Longitude: 40°10’6” E) is among the 5 districts of the Mtwara Region of Tanzania. It is bordered to the south by Mozambique, to the west by the Tandahimba District, to the north by the Lindi Region, and to the east by the Mtwara Urban District and the Indian Ocean. According to the 2012 Tanzania National Census, the population of the Mtwara Rural District was 228,003.^24^ Most of the residents are from the Makonde and Makua tribes. The area of the Mtwara Rural District is 3,597 km^2^, and the district is administratively divided into 6 divisions, 17 wards, and 101 villages.^26^ It has no hospital, but there are 28 dispensaries and 4 health centres. Villages involved in the study included Msijute, Imekuwa, and Naumbu. An estimated 78.8% of the households in Mtwara Rural have at least 1 ITN, and about two-thirds of the population sleeps under mosquito nets.^3^

Kilombero (Latitude: 8°31’ S, Longitude: 37°22’ E) is the name of a river and a district in Morogoro Region, southwestern Tanzania. The district is situated in a vast floodplain between the Kilombero River to the southeast and the Udzungwa Mountains to the northwest. Across the southeast side of the Kilombero River, the floodplain is part of Ulanga District. The population of Kilombero District in 2012 was 407,880.^24^ The main ethnic groups are the Wapogoro, Wandamba, Wabena, and Wambunga. The area is predominantly rural, with the semiurban district headquarters in Ifakara. The majority of villagers are subsistence farmers of maize and rice. Villages visited for the study were Michenga, Mahutanga, Idete, and Ihanga. Mean bed net coverage in Kilombero District was recently estimated to be 44%.^27^

Muheza (Latitude: 5°10’ S, Longitude: 38°46’ E) is among the 8 districts of Tanga Region. It is bordered to the north by Kenya, to the east by the Tanga District and the Indian Ocean, to the south by the Pangani District, and to the west by the Lushoto and Korogwe Districts. In 2012, the population of the Muheza District was 204,461.^24^ Muheza has 1 hospital, 4 health centres, and 44 dispensaries.^28^ The malaria prevalence in the district is about 17%, and the mean bed net coverage is about 39%.^28^ We surveyed the villages of Magila, Ubembe, and Kilulu.

### Participant Recruitment

We conducted 23 KIIs, with village leaders, influential people, opinion leaders, and district health personnel. We purposively selected key informants according to their strategic positions in policy and decision-making processes for malaria prevention and control interventions. In each district, we purposively selected 6 key informants: (1) district malaria control programme coordinator, (2) district medical officer, (3) district executive director, (4) Health Management Information System focal person, (5) Expanded Programme on Immunization focal person, and (6) district health secretary.

We conducted 23 FGDs with separate groups for adult men and women. Eight groups were from Kilombero, 8 were from Mtwara Rural, and 7 were from Muheza. This was done to facilitate more freedom and flexibility during the discussions. Each FGD session consisted of between 5 and 12 participants aged 18 years and older.

### Data Collection

Social scientists from the National Institute for Medical Research facilitated the KIIs and FGDs. The FGDs lasted between 50 and 72 minutes, and the mean duration of the KIIs was 34 minutes. All interviews and FGD sessions were tape-recorded. Unless otherwise stated, all KIIs and FGDs were conducted in Kiswahili, which is Tanzania's official language and is spoken by over 80% of the population. The research team was flexible, however, and allowed participants to express their views using other languages, such as English, if they preferred.

All participants provided oral or written informed consent after receiving an explanation of the study rationale and procedures, including their right to withdraw from the study at any time.

### Data Analysis

The data collected from the FGDs and KIIs were transcribed verbatim by social scientists who were not involved in any of the study's prior activities. We used thematic content analysis, whereby we combined and inductively coded the transcribed notes with handwritten notes composed during KIIs and FGDs. We then used the codes to map out the relationships between themes to produce a thorough synthesis of the data.

To a certain extent, data analysis was an iterative process whereby some initial analysis was conducted concurrently with data collection. When an issue emerged that was not addressed by the interview guide, we added this emerging content to the interview guide and followed it up in the subsequent interviews. The central themes that finally emerged and were included in our analysis were: mechanisms for net distribution, community practices regarding net use and misuse, alternative uses, and disposal practises after net expiry. We did not use computer software for data analysis.

### Ethical Approval

This study received clearance from the National Institution for Medical Research and approval from district executive officers in the participating districts.

## RESULTS

### Net Distribution and Coverage

The majority of participants received their bed nets during bed net distribution activities conducted by the National Malaria Control Programme (NMCP) in 2011. Children under 5 and pregnant women were given first priority during the mass distribution campaigns. In a few instances, elderly villagers were also prioritised. A few participants reported that they bought their bed nets, and a few others stated that they received nets from relatives or friends. Most of the freely distributed nets were of the Olyset and DawaPlus brands. All surveyed districts reported similar varieties of net distribution mechanisms. These included existing supply infrastructures, such as the Expanded Programme on Immunization.

***We have a known structure of distributing nets… and I think it is all uniform in the country because campaigns are always top down. It is the responsibility of the DMOs [district medical officers] to organise which best suit the time and plan of distributing nets in the household. After that, village leaders are mobilised, and they are the ones to organise distribution points, as they well know their administrative boundaries.***

(Female, district health official, 46 years old, Kilombero District, KII)

In all villages, participants considered bed net coverage to be high because each household was reported to have at least 1 bed net. The number of bed nets in each household varied with the number of beds or other sleeping points and family sizes.

***Currently, everyone uses bed nets, unlike old times.***

(Male peasant, 38 years old, Mahutanga Village, Kilombero District, FGD)

***People are now aware: if you don't use a bed net you and your family are in danger. We use the nets.***

(Female, 42 years old, Naumbu Village, Mtwara Rural District, FGD)

According to some participants, for the bed nets that were distributed via a government scheme, whether mass or universal, priority was given to children under 5, pregnant women, and sometimes the elderly:

***First priority… pregnant women then children… especially when bed nets were insufficient… the rest later… but remember that even older persons were sometimes given free nets.***

(Male, district health official, 52 years old, Muheza District, KII)

### Bed Net Preferences

Preferences for specific bed net types and brands depended on their perceived effectiveness. The main issue that was singled out by many interviewees and FGD participants was the nets' ability to prevent mosquito bites and malaria, which was considered a major cause of morbidity and mortality in many households. Many participants had a common perception that Olyset bed nets have larger mesh spaces, which allow mosquitoes to penetrate. Bed net quality, associated by participants mainly with how easily a net can be torn, was also a critical factor influencing preference. The majority of key informants and FGD participants reported that some net brands were undesirable because they could easily be torn to create holes for easy penetration by mosquitoes.

Participants reported that bed net colour preferences, particularly blue and green, were greatly influenced by availability in their villages. Suppliers, especially those distributing free or subsidised bed nets, often provided blue and green bed nets. Some participants preferred coloured nets because they are not easily soiled or stained with dirt and dust and, therefore, do not need to be washed as frequently as white bed nets.

***People prefer green nets… they do not like white nets because of the issue of cleanliness…hence you find most nets here are blue and green.***

(Male, 40 years old, Magila Village, Muheza District, FGD)

### When do People Stop Using Nets?

During FGDs, varied opinions were expressed regarding when to stop using nets. Some participants stated that people should stop using nets when they are worn out and during seasons when mosquito numbers are low.

***From August until this December, if you walk to all households, nets are hanged – no nets are used.***

(Male, 49 years old, Msijue Village, Mtwara Rural District, FGD)

### Alternative Uses of Bed Nets

Participants reported several alternative uses of bed nets seen in their villages, including making fences, protecting chickens from predators, and fishing activities. Very few bed nets were regarded as waste in the participating communities. Participants from Mtwara reported particularly diverse uses; old nets were used as shades for vegetable gardens and others fitted as latrine walls.

Most FGD participants from Muheza District agreed that totally worn-out nets should be thrown away or burned because they were useless.

***Those nets you see in the garbage [pointing to discarded nets] cannot serve any useful purpose. You can neither use them for sleeping under nor for any beneficial alternative.***

(Male, district health official, 41 years old, Muheza District, KII)

Participants believed in other personal protection measures as the best strategies to prevent malaria. Among these were using treated damaged nets as curtains.

***Normally, damaged nets have bigger holes and are ineffective for preventing entry by mosquitoes; they, however, can function better when used as curtains.***

(Male, 33 years old, Magila Village, Muheza District, FGD)

### Common Practices for Bed Net Disposal

Several net disposal practices existed in the participating communities. The most commonly reported practices were disposing of or burning worn-out bed nets along with other household waste. In villages participating in free net distribution campaigns, residents reported being advised to pack expired nets into bags before returning them to collection teams.

### Bed Net Collection

In view of the perceived environmental and health risks associated with bed net disposal, participants described alternative, organised bed net removal strategies. As long as the removal was accompanied by provision of new or better bed nets, participants reported that communities were ready to hand over even relatively intact nets.

Community preferences for net collection included designating a specific drop-off location within the community and assigning an organisation to facilitate the optimal collection and replacement strategies based on community members' input and needs. FGD participants and key informants unanimously agreed that – according to how the administrative system is organised – wherever bed nets must be collected, health facilities and village offices are appropriate collecting venues, and health facility management and village leaders should supervise the process.

***We have agreed here that waste nets that are no longer in use should be collected, how?… We have health centres, dispensaries, and village offices – these are the best places where nets not in use may be collected. Leaders of these institutions should lead the exercise, but for those living far away from these centres, we have leaders within their localities who may help with collecting and bringing waste nets to the proposed collection centres.***

(Male, district health official, 52 years old, Kilombero District, KII)

### Community Acceptability of Bed Net Removal and Recycling

Although community members were willing to surrender their bed nets for recycling, incentives – either in the form of monetary compensation at the price of a new net or a net replacement – were mentioned as expectations or necessities. Some participants contended that a lack of compensation or failure to replace old nets could lead to reduced bed net usage. Moreover, it was stated that collectors encounter difficulties and resistance when bed net collection is arranged without the intention of replacement or compensation.

***People won't be happy… it will affect the net usage negatively, as not all people will be able to buy their own nets, and it will be bad, as people will have started to protect themselves and suddenly nets are taken away … there will be nothing to motivate them.***

(Female, 36 years old, Idete Village, Kilombero District, FGD)

### Bed Net Recycling and its Association With “Good” Alternative Uses

Due to low levels of knowledge among community members about the best alternative uses and disposal methods for bed nets, as well as limited alternative use options for worn-out nets, many participants did not associate net recycling with “good” alternative uses. They insisted that nets are properly used when they are new and capable of protecting people from mosquito bites and nuisance; only after bed nets are used for protection against mosquitoes should they then be relegated to an alternative use. However, nets that were too worn out to protect users from mosquitoes were often considered too worn out for alternative uses.

***A net is assigned another use, for example fishing or protecting chicks from predators, only when users convince themselves that it can no longer protect them from mosquito bites.***

(Male, district health official, 43 years old, Muheza District, KII)

There was general agreement among participants that it is abnormal for new bed nets to first be used for purposes other than mosquito protection and malaria prevention, only to later fulfil their intended function.

***A new net cannot be assigned alternative uses because somehow people are aware of the importance of using nets for protecting household members from mosquito bites and malaria. In addition, many people cannot afford to buy a new net every time they need one – because of poverty… so they rely on those nets which are distributed for free or those obtained through a subsidised voucher system [Hati Punguzo]… In this regard, it is very unlikely to expect a new net to first be used for alternative purposes and later be used as a bed net proper.***

(Male, 44 years old, Kilulu Village, Muheza District, FGD)

### Risks and Benefits of Alternative Uses of Old Bed Nets

The participants reported a diverse set of perceptions about the environmental and health-related risks and benefits associated with alternative uses of old nets, with consensus among participants predominating over disagreement. Most key informants and FGD participants reported that the perceived lack of risk was associated with a lack of adequate knowledge among community members about health and environmental hazards associated with alternative uses of bed nets.

***What most people know, and especially what they hear from the radios, is that nets will protect them from mosquito bites and, thus, malaria.… Even in health facilities, we have not seen anything educating us on how to keep nets after they are no longer used as mosquito protectors… we have not gotten any information regarding the relationships between net use, health, and the environment… So it is difficult for community members to stop doing “business as usual.” They will keep disposing of old nets conventionally in garbage pits or burn them, as they do with other household waste.***

(Female, district health official, 37 years old, Mtwara Rural District, KII)

Alternatively, many participants asserted that community members might be aware of the potential environmental and health risks but are not informed of the best ways to deal with old nets as special waste. Participants reported that most community members believed that discarding nets in garbage pits or burning them like any other type of waste are the normal and preferred disposal methods.

A few key informants claimed that some community members were aware of the health and environmental risks associated with alternative uses and improper disposal of used nets and emphasised the community's perception that:

***Materials used to manufacture most of the ITNs can hardly be decomposed and thus pose an environmental threat to the soil ecology.***

(Male, district health official, 54 years old, Muheza District, KII)

### Community Perceptions of the Presence of Insecticides in Bed Nets

Participants expressed worries about the presence of insecticides in bed nets and their carrier bags. Most commonly, FGD participants across all sites wondered if the insecticides that impregnated the nets can kill mosquitoes and others insects, what were their effects on humans, especially if people inhale these chemicals? Such worries and potential hazards of ITN use led participants to call for authorities to conduct effective community sensitisation and health education campaigns to address these issues.

***These bed nets have chemicals that kill mosquitoes and other insects, and we are told that they can last for about 5 years. Our worry is that sometimes the bed nets' plastic bags have further domestic reuse. What will now happen to the users of these bags if they are not properly disposed of?***

(Male, 49 years old, Ihanga Village, Kilombero District, FGD)

## DISCUSSION

Malaria still poses a threat to public health and socioeconomic well-being among populations in endemic countries. Over time, ITNs gradually become less effective and worn, and must be regularly re-treated or replaced. This study sought to investigate ITN use and misuse among residents of 3 districts in Tanzania, which were selected because of their high rates of ITN coverage. We also explored perceptions related to net expiry and disposal, the associated environmental and health impacts, and community acceptability of alternative disposal strategies.

### Net Coverage and Usage

Bed net coverage and usage rates were high in the participating communities, especially among pregnant women and children under 5. This was partly attributable to the general bed net distribution that is organised by the NMCP that had taken place just prior to the study period. Additionally, the NMCP, through the National Voucher Scheme that was initiated in Tanzania in 2004, has targeted pregnant women and their infants for subsidised ITN allocations via voucher distribution at antenatal clinics.

Our study findings suggest that appropriate net usage and coverage may be enhanced by the presence of effective collection and waste disposal mechanisms. The majority of our study participants emphasised that organised incentive-based systems for the removal or collection of old nets would be used if people were assured that they would receive new nets; they would have no reason to continue hanging worn-out and tattered ITNs.

### Community Misconceptions Regarding ITN Use

Several studies have reported fears and misconceptions associated with ITN use, particularly related to potential harm caused by the chemicals used to treat the nets.^29–31^ Many participants expressed worry about the consequences of using ITNs in extreme heat, and some participants were concerned about a possible link between ITN use and impotence or infertility. Participants also reported mild side effects, such as skin rashes, among people who might have been allergic to the insecticide. Studies elsewhere have demonstrated that sociocultural beliefs among community members have important bearings on peoples' decisions to use or not use bed nets.^23,29,30^ To dispel these fears and misconceptions, it should be made clear to all bed net users and recipients that the chemicals used for ITNs kill and repel mosquitoes but are safe for humans when used correctly.

### Bed Net Preferences

A key policy implication suggested by these findings is that authorities should design and implement strong and effective quality control and monitoring strategies to make sure that manufacturers produce nets of the required standard, which appeal to not only those who pay for production and distribution but also to end users.

Our findings revealed that individual households may have had up to 3 or more new and unused nets, which did not match bed sizes or were considered incompatible with household sleeping arrangements.

### Bed Net Disposal

International and national malaria control stakeholders have emphasised increasing bed net coverage and usage among populations at risk of malaria infection.^4,18^ However, there is limited empirical evidence on net disposal practices in communities that benefit from malaria control interventions, such as bed net distribution. Furthermore, there are no comprehensive international or national policy guidelines regarding what community members are supposed to do with worn-out ITNs. The majority of community members in our study were not aware of how to properly dispose of used nets, leading them to improper disposal practices associated with environmental and health risks through pollution.

### Bed Net Recycling and Its Association With “Good” Alternative Uses

Bed net recycling has been demonstrated as a feasible and needed option for sustainable management of long-lasting insecticidal nets, especially when they have lost their efficacy in protecting humans from mosquito bites and malaria infection.^4,5^ This study demonstrated that community members are willing to participate in efforts to maximise appropriate alternative uses of old ITNs, and the findings have called attention to the fact that ongoing mosquito net campaigns have not seriously considered packaging information about the proper management of used nets. Participants reported a low level of knowledge about the best ways to dispose of nets or reuse them for other purposes.

Moreover, there were reportedly limited options regarding what nets should be used for after they are declared waste. Because of this shortcoming, the majority of participants did not associate net recycling with “good” alternative uses, especially after nets were declared as waste. Like many countries in sub-Saharan Africa, Tanzania lacks adequate waste disposal infrastructure, and the existing waste management laws are either weak or lack adequate enforcement mechanisms. It is, therefore, difficult to institutionalise incentives to motivate widespread sensitisation about health and environmental issues related to proper management of waste, including used bed nets.
